# Measles Virus Infection and Immunity in a Suboptimal Vaccination Coverage Setting

**DOI:** 10.3390/vaccines7040199

**Published:** 2019-11-28

**Authors:** Monia Pacenti, Nataskya Maione, Enrico Lavezzo, Elisa Franchin, Federico Dal Bello, Lorena Gottardello, Luisa Barzon

**Affiliations:** 1 Microbiology and Virology Unit, Padova University Hospital, 35128 Padova, Italy; monia.pacenti@aopd.veneto.it (M.P.); elisa.franchin@unipd.it (E.F.); federico.dalbello@unipd.it (F.D.B.); 2 Department of Molecular Medicine, University of Padova, 35121 Padova, Italy; nataskyamaione@yahoo.it (N.M.); enrico.lavezzo@unipd.it (E.L.); 3 Department of Hygiene and Public Health, Azienda ULSS 6 Euganea, 35131 Padova, Italy; lorena.gottardello@aulss6.veneto.it

**Keywords:** measles, epidemiology, vaccine uptake, vaccine hesitancy, cross-protection, whole-genome sequencing, secondary vaccine failure, neutralizing antibody, outbreak, immunity

## Abstract

Despite efforts to improve surveillance and vaccination coverage, measles virus (MeV) continues to cause outbreaks also in high-income countries. As the reference laboratory of the Veneto Region, Italy, we analyzed changes in population immunity, described measles outbreaks, investigated MeV genetic diversity, and evaluated cross-protection of measles vaccination against MeV epidemic strains. Like most European areas, the Veneto Region has suboptimal measles vaccination coverage and is facing a growing public mistrust of vaccination. A progressive decline of measles vaccine uptake was observed during the last decade in the Veneto Region, leading to immunity gaps in children and young adults. Measles outbreaks were caused by the same MeV genotype B3, D4, and D8 strains that were circulating in other European countries. Eleven cases of measles were observed in immunized subjects. These cases were not associated with particular MeV genotypes nor with mutations in epitopes recognized by neutralizing antibodies. Accordingly, sera from fully vaccinated subjects cross-neutralized epidemic MeV strains, including the genotypes B3, D4, and D8, with the same high efficiency demonstrated against the vaccine strain. In fully vaccinated subjects, high MeV IgG antibody titers persisted up to 30 years following vaccination. These results support the use of the current measles-containing vaccines and strategies to strengthen vaccination.

## 1. Introduction

Despite efforts to improve surveillance and vaccination coverage, measles virus (MeV) continues to cause outbreaks not only in low-income but also in high-income countries [1]. During the last two years, Europe and neighboring countries reported a dramatic increase in the number measles cases, with 25,475 and 59,578 cases reported in 2017 and 2018, respectively, by the 53 countries of the World Health Organization (WHO) European region, compared with the 5273 measles cases reported in 2016 [2]. Among European countries, Romania, Italy, Greece, France, and the United Kingdom have been the most affected, all characterized by a vaccination coverage below the 95% target defined by WHO for measles elimination [3]. The increasing distrust of vaccination among people represents an important obstacle to the success of routine vaccination programs [4,5]. Combined with a decrease of the natural immunity of the population and waning antibody titers in vaccinated people, a low vaccination coverage generates immunity gaps in specific age groups, who remain susceptible to infection and can sustain transmission during outbreaks, as recently predicted by a modelling analysis [6]. Another factor that has been hypothesized to account for the current resurge of measles is the emergence of new viral strains characterized by increased transmissibility and escape from vaccine-induced immunity [7]. Actually, the recent and ongoing measles outbreaks have been mainly caused by two MeV genotypes, i.e., genotype D8, which has been circulating in Europe during the last decade, and a genotype B3 strain, which has been introduced in Europe more recently [8]. This B3 strain has higher transmissibility than other MeV genotypes [8,9], is highly virulent in a macaque model [10], and has been shown to be neutralized less efficiently than other MeV genotypes by sera of vaccinated individuals [11].

In line with the WHO 2012–2020 Global Measles and Rubella Strategic Elimination Plan and the Midterm Review, which recommended research on susceptibility profiles for measles and research related to outbreaks in high-vaccine-coverage settings [12], the aim of this study was to investigate multiple aspects of measles infection and population immunity by an integrated analysis of field and experimental data obtained during the last decade in the Veneto Region, Italy, as a representative region in Europe. More specifically, we analyzed changes in population immunity during the last two decades in the Veneto Region, described measles outbreaks and cases of MeV infection among supposedly immunized patients, investigated MeV genetic diversity within transmission clusters, and evaluated if measles vaccination provided cross-protection against MeV epidemic strains. The Veneto Region, located in the northeastern part of the country, is one of Italy’s richest regions. The health of the Veneto Region’s resident population of 5 million inhabitants compares favorably with that of the population of other regions in Italy and within Europe [13]. Like other European countries, this Region is facing the problem of growing public mistrust of vaccination and a decline in vaccine uptake, which increase the risk of population exposure to vaccine-preventable infectious diseases like measles [14,15].

## 2. Materials and Methods

### 2.1. Study Design

All the investigation reported in this study were performed at the Subnational Reference Laboratory of the Measles and Rubella surveillance in the Veneto Region, following routine procedures according to the national and regional measles surveillance and elimination plans.

#### 2.1.1. Seroprevalence Study

To determine local MeV seroprevalence, we retrieved the results of MeV antibody screenings routinely performed in all employees at the University of Padova and Padova University Hospital for preventive medicine evaluation. Age range of the subjects was 20–65 years. Two time intervals were considered, i.e., January 2009 to December 2012 and January 2014 to December 2017.

#### 2.1.2. Measles-Specific Antibody Titers in Vaccinees

In a subgroup of the above subjects, for whom records on measles vaccination were available, MeV IgG antibody titers determined by enzyme immunoassay (EIA) were analyzed according to the time interval since the second measles vaccine dose.

#### 2.1.3. Measles Cases

Specimens of serum, urine, and throat swabs collected from suspected cases of measles in the Veneto Region were referred to the Subnational Reference Laboratory of the Veneto Region for laboratory confirmation. Measles cases with onset of symptoms between January 2010 and December 2018 were included in the study.

#### 2.1.4. Measles Vaccine Cross-Neutralization Study

To investigate if antigenic differences between the vaccine strain and the wild-type MeV epidemic strains might result in reduced efficacy of measles vaccination, we compared the titers of neutralizing antibodies against the MeV Edmonston A Schwarz vaccine strain and the MeV genotype B3, D4, and D8 strains, which were isolated during the outbreaks, in the sera collected from a group of asymptomatic individuals aged 18–25 years, who previously received two doses of measles-containing vaccine and had MeV IgG antibody titers ranging from 0.300 to 0.700 IU/mL.

### 2.2. Laboratory Procedures

Cases were confirmed by the demonstration of MeV-specific IgM antibodies in serum or by the detection of MeV RNA in nasopharyngeal swabs and urine specimens. Laboratory tests were performed at the Subnational Reference Laboratory for Measles and Rubella in the Veneto Region (Microbiology and Virology Unit of Padova University Hospital), which is an accredited member of the Network of Italian Reference Laboratories for Measles and Rubella (MoRoNet). MeV RNA testing was performed by in house real-time RT-PCR. MeV-specific IgM and IgG antibodies and avidity of MeV IgG antibodies were tested with the Enzygnost anti-Measles IgM e IgG kit (Siemens Healthcare Diagnostics, Erlangen, Germany). MeV genotypes were determined by sequencing the 450 nucleotides encoding the carboxyl-terminus of the N gene, according to WHO guidelines [16].

Sequencing of the full MeV genome and the MeV hemagglutinin (H) gene was performed by the Sanger method on 22 overlapping amplicons of about 500–700 bp and 9 overlapping amplicons of about 300 bp, respectively.

MeV was isolated from clinical samples by inoculation onto B95-A cells and titrated by the tissue culture infectious dose 50 (TCID50) method in 96-well plates. The neutralizing activity of antibodies induced by measles-containing vaccine was evaluated by microneutralization assays with the Edmonston A Schwarz measles vaccine strain and clinical isolates of MeV in B95-A cells grown in 96-well plates. The 50% neutralizing dose (ND50) was calculated with the Karber formula. A neutralizing titer <1:8 was considered negative.

### 2.3. Evolutionary and Phylogenetic Analyses

For evolutionary analysis, the most abundant B3 dataset (25 complete genomes) was input to BEAST [17] together with the corresponding sampling dates. The mutation rate was calculated using the general time-reversible (GTR) model [18], a relaxed molecular clock, and a coalescent/exponential growth tree prior. The phylogenetic results were further confirmed by repeating the analysis with different substitution models and prior and molecular clock parametrizations.

The dN/dS ratio was calculated through the Datamonkey webserver [19], using the fixed effects likelihood (FEL) and fast unconstrained Bayesian approximation (FUBAR) package [20,21]; sufficient diversity for the analysis was present only in the nucleoprotein (N) and large polymerase protein (L) genes of both B3 and D8 genotypes and for H in the D8 genotype.

A comprehensive phylogenetic analysis of MeV strains detected during outbreaks was performed with MEGA 7 [22] on the MeV N 450-nt carboxyl-terminal sequence. The neighbor-joining method was applied [23].

### 2.4. Statistical Analysis

Continuous variables were reported as mean ± standard deviation, categorical variables were summarized as numbers and percentages. Neutralizing antibody titers were reported as geometric mean and confidence interval (CI) of 95%. Comparisons between continuous variables were carried out by two-tailed unpaired *t*-test; comparisons between categorical variables were carried out by χ^2^ test; comparisons of antibody titers were performed in Log-transformed values by two-tailed unpaired *t*-test and Pearson’s correlation. Statistical significance was determined by a *p* value of less than 0.05. All analyses were performed using Statistica version 14 (Dell, Round Rock, TX, USA) and Graph-Pad Prism version 8 (GraphPad Software, San Diego, CA, USA).

## 3. Results

### 3.1. Measles Vaccination Uptake and Seroprevalence

Decreased population immunity and low vaccine uptake due to hesitancy of the population toward vaccination represent key factors for the resurgence of measles epidemics in industrialized countries. In Italy, measles vaccination is recommended since 1979, but the Veneto Region suspended mandatory vaccination since January 2008. To evaluate if the suspension led to changes in population protection against measles during the last decade, we analyzed data on measles vaccination uptake and seroprevalence. Data on measles vaccination uptake were retrieved from reports of the Ministry of Health [24] and the Veneto Region [25], while data on MeV seroprevalence were obtained by a retrospective analysis of the results of routine MeV antibody testing in employees of the University of Padova and Padova University Hospital for preventive medicine evaluation. This cohort was not representative of the whole Veneto Region because it included mainly people from Padova province. However, since vaccination coverage in Padova is within the average of the Region, the investigated cohort can be considered an acceptable approximation of the situation in the Veneto Region.

**Figure 1:**
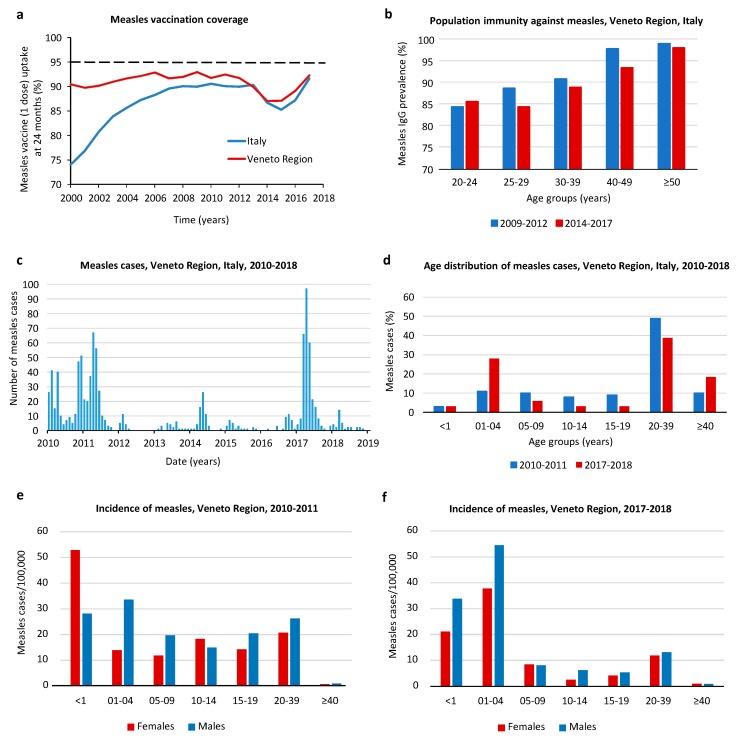
Measles vaccination coverage and seroprevalence. (**a**) Percentage of uptake of one dose of measles-containing vaccine in children aged 24 months in the Veneto Region and Italy during the period from 2000 to 2017. (**b**) Prevalence of measles-specific IgG antibodies in employees of the University of Padova and Padova University Hospital, Veneto Region, Italy. Two periods (2009–2012 and 2014–2017) were compared. (**c**) Epidemic curve of 1005 measles cases in the Veneto Region, Italy, from January 2010 to December 2018. (**d**) Distribution of measles by age group during the 2010–2011 and the 2017–2018 outbreaks in the Veneto Region, Italy. (**e**) Incidence of measles by age and sex groups during the 2010–2011 and (**f**) the 2017–2018 outbreaks in the Veneto Region, Italy.

In the Veneto Region, during the last decade, the mean coverage rate for the first dose of measles-containing vaccine in children aged 24 months ranged from 87.1% to 93% in the different years (Figure 1a). A progressive reduction of vaccination coverage was observed since 2008, which was, however, in line with the national trend (Figure 1a). As a response to the decreased vaccination uptake, the Italian Parliament approved law n. 119 in July 2017, augmenting the mandatory childhood vaccines from 4 to 10, including mandatory measles vaccination [26]. This led to a slight increase of measles vaccination uptake, which, however, remained below the 95% coverage target defined by the WHO measles elimination plan (Figure 1a).

Analysis of measles immunity was conducted on 11,506 subjects who were screened for MeV IgG antibodies in the 2009–2012 period and on 9892 subjects tested in the 2014–2017 period (Figure 1b). The comparison of the data from the two periods showed a trend toward a general decrease in population immunity. Population protection with seroprevalence over 95% was observed only in groups of subjects older than 40 years (period 2009–2012) and 50 years of age (period 2014–2017), mainly representing people with naturally acquired immunity. In the other age groups, measles IgG seroprevalence ranged from 74% to 89%.

### 3.2. Description of Measles Outbreaks

In the Veneto Region (population of about 5 million people), 1005 measles cases were reported in the period from January 2010 to December 2018. During this period, large outbreaks occurred in 2010–2011, with 465 measles cases reported (66% were laboratory-confirmed), and in 2017–2018, with 322 measles cases (86% were laboratory-confirmed) (Figure 1c). In Italy, during the same period, 20,746 measles cases were notified, with peaks of incidence in 2010–2011, 2013–2014, and 2017–2018 (source EpiCentro, Istituto Superiore di Sanità [27]). In the Veneto Region, as at the national level, most measles cases were unvaccinated subjects (82.8%), 9.9% received only one dose of measles-containing vaccine, and 2.0% received two doses; information on vaccination status was not available for 5.2% of cases. The median age of the cases was 25.5 years (range 0–69 years) in both outbreaks. Children aged ≤4 years and young adults aged 20–39 years were the most affected age groups (Figure 1d). The estimated annual incidence of measles was 5.9 cases/100,000 inhabitants in the general population and 45 cases/100,000 in children ≤4 years of age. Overall, 58% of measles cases were males, and the incidence of measles was slightly higher in males than in females (Figure 1e,f). In the 2010–2011 and 2017–2018 outbreaks, 23% and 34% of patients, respectively, were hospitalized; complications included kerato-conjunctivitis (16%), diarrhea (15%), pneumonia (10%), laryngo-tracheo-bronchitis (9%), respiratory distress (6%), otitis (5%), and hepatitis (2%). Abortion occurred in a young female with measles and without other risk factors. No measles-related deaths were reported in the Veneto Region.

**Figure 2:**
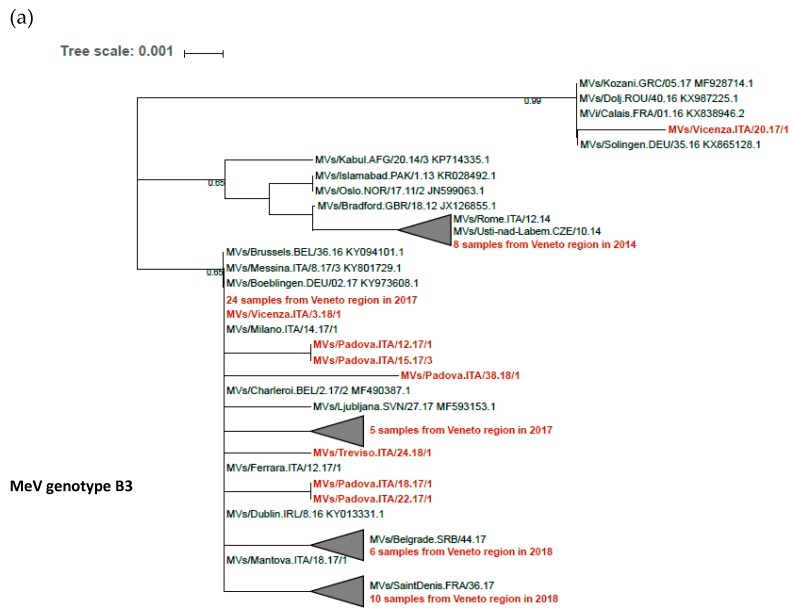

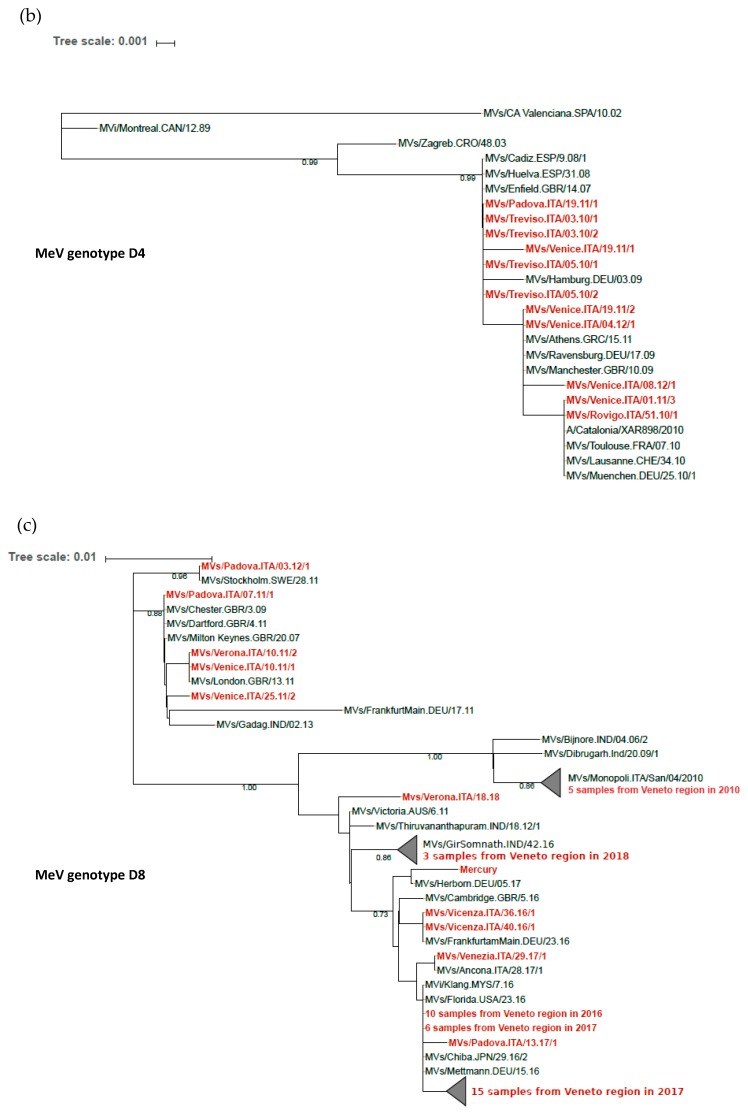
Phylogenetic trees of MeV sequences obtained from measles cases, Veneto Region, 2010–2018. MeV genotypes B3 (**a**), D4 (**b**), D8 (**c**). Phylogenetic analysis was performed with MEGA 7 [22] on the 450-nt carboxyl-terminal sequence of the MeV nucleoprotein (*N*) gene, which was available for 61, 11, and 50 samples of B3, D4, and D8 genotypes, respectively. The neighbor-joining method was applied [23].

MeV genotypes D4 and D8 co-circulated in the Veneto Region during the 2010–2011 outbreak and accounted for 53% and 45% of sequenced MeV strains, respectively, while MeV genotypes B3 (72%) and D8 (28%) co-circulated during the 2017–2018 outbreak. Within these MeV genotypes, phylogenetic analysis showed the presence of different variants in transmission clusters, which is consistent with multiple introductions of the virus (Figure 2).

### 3.3. MeV Genome Diversity and Evolution in Transmission Clusters

**Figure 3:**
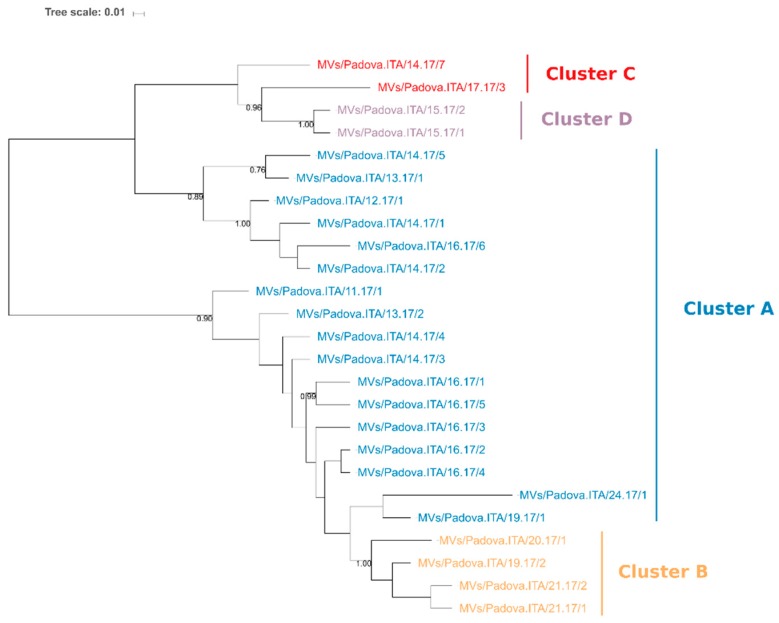
Phylogenetic tree of whole genome sequences of MeV genotype B3 from patients involved in transmission clusters. Bayesian maximum clade credibility tree, obtained using the Bayesian Evolutionary Analyses Sampling Trees (BEAST v 1.10.4) software [17]. The software was run with the general time-reversible substitution model, a gamma distribution for the rate variation among sites, an exponential relaxed clock, and a coalescent/exponential growth tree prior. Posterior support values above 70% are displayed. Transmission clusters identified by epidemiological investigation are indicated as A to E and highlighted with different colors.

To investigate the genetic diversity and evolution of MeV during an outbreak, we sequenced the MeV full genome directly in biological samples collected from subjects involved in transmission clusters, according to epidemiological links. A total of 25 full genome sequences were obtained from five transmission clusters of MeV genotype B3 that occurred in 2017, including a large transmission cluster (Cluster A) which started from contact in an emergency room (Figure 3). In addition, five full genome sequences were obtained from two transmission clusters of MeV genotype D8 during 2017.

The mutation rate of MeV B3 and D8 was estimated as 7 × 10^−4^, in agreement with the literature on MeV and on RNA viruses in general [28]. No positive selection pressure was identified at any site in the viral genome (Table 1).

Phylogenetic and evolutionary analysis of MeV B3 full genome sequences identified relationships that did not fully correspond to the transmission clusters identified by epidemiological investigation: e.g., transmission cluster A included two different evolutionary groups (Figure 3).

### 3.4. Vaccine-Induced Cross-Neutralizing Antibodies against Epidemic MeV Strains

**Figure 4:**
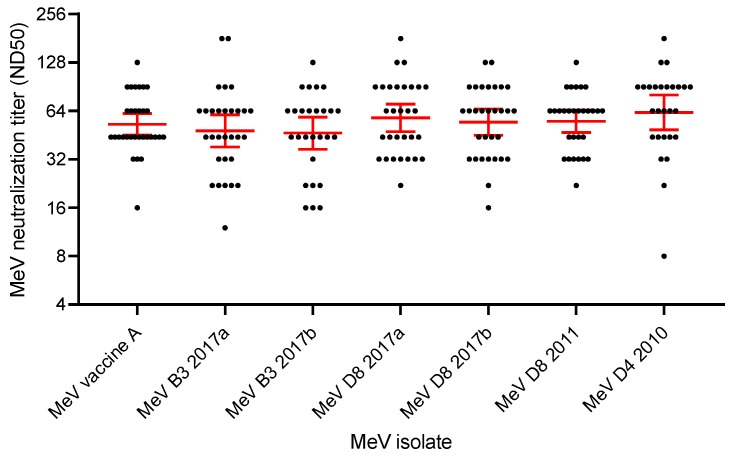
Vaccine-induced cross-neutralization of epidemic MeV strains. Cross-neutralizing antibody titers against epidemic MeV strains were measured in serum samples obtained from subject who had received two doses of measles-containing vaccine. Serum samples were selected according to MeV IgG titers measured by enzyme immunoassay, in order to obtain five groups, each containing six samples with homogeneous MeV IgG titers of approximately 0.300 IU/mL, 0.400 IU/mL, 0.500 IU/mL, 0.600 IU/mL, and 0.700 IU/mL, respectively. The following MeV isolates were used in microneutralization assays: MeV vaccine strain Edmonston genotype A, two MeV genotype B3 strains isolated in the Veneto Region in 2017, one MeV D4 strain isolated in 2010, one MeV D8 strain isolated in 2011, and two MeV D8 strains isolated 2017. Neutralization titers are reported as 50% neutralizing dose (ND50) values. Geometric mean values and 95% confidence intervals are indicated in red.

The results of MeV neutralization assays in a group of healthy subjects vaccinated with two doses of measles-containing vaccines showed that all subjects had neutralizing antibodies against the MeV vaccine strain and the clinical isolates of epidemic MeV genotype B3, D4, and D8 strains, without significant differences in neutralization titers among sera with different levels of MeV IgG antibodies. In addition, the cross-neutralizing antibody titers against the epidemic MeV strains were not significantly different from the neutralization titers against the vaccine strain (Figure 4).

**Figure 5:**
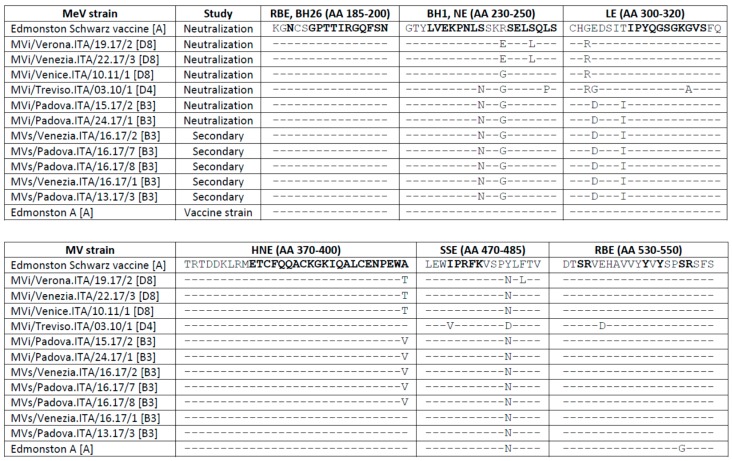
Analysis of hemagglutinin protein epitopes in epidemic MeV strains. Alignment of the amino acid sequences of hemagglutinin protein epitopes of the MeV epidemic strains that were used in the neutralization assays and the reference MeV Edmonston A vaccine strain (indicated as Neutralization) and of MeV sequences obtained from cases of measles in previously immunized individuals (indicated as Secondary). The amino acid sequence of the prototype Edmonston A vaccine strain is also shown. The epitopes recognized by neutralizing antibodies are highlighted in bold. Amino acid variants compared to the reference MeV Edmonston A vaccine strain are shown. AA: amino acid; RBE: receptor-binding epitope; BH26, BH1: epitope recognized by the BH26 neutralizing antibody; NE: neutralizing epitope; LE: loop epitope; HNE: hemagglutinating and noose epitope; SSE: sugar-shielded epitope.

We also analyzed if mutations had occurred in neutralizing epitopes of MeV strains involved in the outbreaks. MeV possesses two glycoproteins on the envelope, the H and fusion (F) proteins. The H glycoprotein is involved in binding to cellular receptors on the target host cells, which triggers F protein-mediated membrane fusion and virus entry into the cell. Following infection or vaccination, neutralizing antibodies directed against both H and F glycoproteins are elicited, but those targeting the H glycoprotein mainly account for protection against MeV infection [29]. Thus, we sequenced the full gene encoding the H glycoprotein and compared the amino acid sequence of the H protein of MeV isolates used in the cross-neutralization assay, which were representative of outbreak strains, with the sequence of the Edmonston A vaccine strains. This analysis showed that the putative epitopes targeted by the neutralizing antibodies (i.e., BH1, NE, HNE, SSE, RBE, and BH26 epitopes) were conserved in epidemic MeV strains (Figure 5).

### 3.5. Measles in Previously Immunized Subjects

Eleven cases of MeV infections in previously immunized subjects occurred during the 2017–2018 outbreak, including five subjects who received two doses of measles-containing vaccine, according to the immunization records of the Veneto Region (median time since last vaccine dose, 22 years; range 9–26 years), and six subjects for whom records on vaccination were not available (Table 2). At the time of diagnosis, these measles cases had low or absent MeV IgM antibodies, high titer and high avidity of MeV IgG antibodies, and MeV RNA detectable in body fluids. In these patients, MeV RNA loads in nasopharyngeal swabs and in urine were significantly lower than in a control group of 40 unvaccinated subjects with primary measles, who were selected based on matching for age and time since the onset of symptoms (nasopharyngeal swab, mean C_T_ value 27.6 ± 4.8 vs. 22.7 ± 5.5, respectively, *p* < 0.05; urine, mean C_T_ value 31.4 ± 7.7 vs. 22.8 ± 4.5, respectively, *p* < 0.01). The MeV genotype distribution in cases with previous measles immunity was similar to that in the general population (Table 2).

To assess if these measles infections were caused by MeV strains with mutations in amino acid residues targeted by neutralizing antibodies, we sequenced the MeV *H* gene directly from biological samples. Sequencing of the full MeV B3 *H* gene was obtained from a patient who was previously vaccinated with two vaccines doses and from four subjects with unknown vaccination status. In all these cases, no amino acid changes were identified in the H protein compared to the H protein of the MeV B3 and D8 strains responsible for primary infections (Figure 5).

### 3.6. Persistence of Measles Vaccine-Induced Immunity

**Figure 6:**
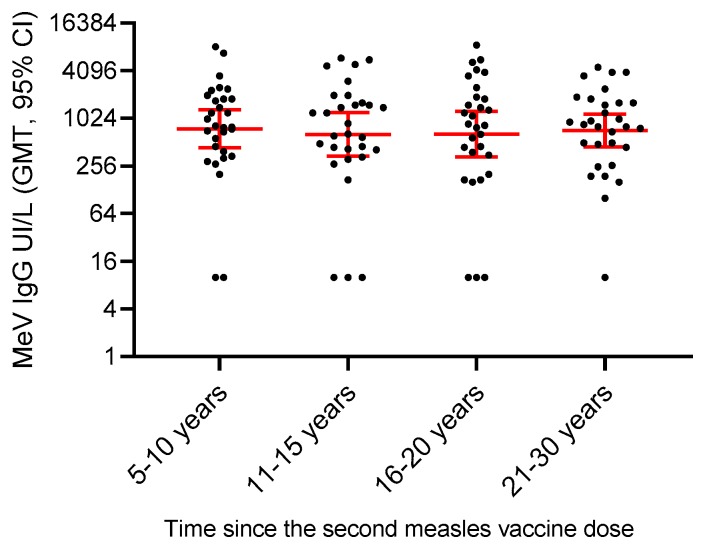
Persistence of MeV antibodies in vaccinated individuals. MeV IgG antibody levels, reported as IU/L, were determined by enzyme immunoassays in subjects who received ≥2 doses of measles-containing vaccine (the groups included 30 subjects for each time interval since the last vaccine dose). Geometric mean values and 95% confidence intervals are indicated in red.

To evaluate the persistence of measles vaccine-induced immunity, the titers of MeV IgG antibodies were analyzed in a group of subjects for whom information on measles vaccination status was available and who had received ≥2 doses of measles-containing vaccine. This analysis showed the presence of protective MeV IgG antibody titers (i.e., >0.120 UI/mL) in over 90% of subjects and the persistence of sustained antibody levels up to 30 years after vaccination (Figure 6), in agreement with previous reports [30].

## 4. Discussion

This study presents an integrated analysis of field and laboratory data on measles infection and population immunity in a typical European setting characterized by suboptimal vaccination coverage and the occurrence of relatively large measles outbreaks. In this context, this study analyzed changes in vaccination coverage and population immunity, described the clinical features and the molecular epidemiology of the measles outbreaks, investigated the genetic diversity and evolution of MeV within transmission clusters and the emergence of potential escape mutants, especially in cases of secondary vaccine failure, and, finally, it evaluated if measles vaccination provided cross-protection against epidemic strains of MeV genotypes B3, D4, and D8. The results of this study from field data provide a coherent picture that supports strengthening vaccination programs for measles eradication.

Analysis of measles vaccination coverage, population immunity from serological surveys, and the demographics of measles outbreaks during the last decade in the Veneto Region identified immunity gaps in children aged 0–4 years and in young adults aged 20–39 years and a trend towards a decrease of measles immunity among young children and subjects older than 40 years. This trend is the result of the progressive reduction of vaccine uptake observed during the last decade, added to the lower circulation of the virus. These findings are partially in agreement with the results of a recent modelling analysis, which predicted an increase of the susceptible population among individuals older than 15 years, but no relevant changes in measles susceptibility in children aged 0–4 years in Italy, because it assumed that vaccination schedules and coverage remained unchanged [6]. The incidence of measles was higher in males than in females, but differences were small and not consistent in different age groups and years. In the literature, severe complications of measles have been reported more frequently in males than in females [31], and slightly higher antibody levels induced by measles vaccination have been observed in girls than in boys [32]. However, data in the literature are very limited, and further studies stratifying data according to gender are needed to clarify the relevance of sex for vaccine-induced protection and for the risk of severe disease.

Measles outbreaks that occurred in the Veneto Region during the last decade were caused by the same MeV genotype B3, D4, and D8 strains that were circulating in other Italian Regions and European countries. Measles is a highly contagious infectious disease, and MeV strains rapidly circulate with traveling people, causing disease globally, such as in the case of the recently emerged MeV genotype B3 strain [2]. To get a more detailed picture of measles transmission networks, we sequenced the full viral genome from clinical samples collected from subjects involved in different transmission clusters. A phylogenetic analysis identified transmission patterns different from those indicated by conventional epidemiological investigation, thus supporting the utility of full viral genome sequencing for the epidemiological investigation of measles outbreaks [33].

During the 2017–2018 outbreak, we observed 11 cases of measles in previously immunized subjects, probably representing cases of secondary vaccine failure. Measles infections in vaccinated individuals are increasingly being reported when outbreaks occur in highly vaccinated populations [24,25,26,27,28,29,30,31,32,33,34,35,36]. Like for other cases reported in the literature [34,35,37], the symptoms were milder, and the viral RNA loads in body fluids were significantly lower than in measles-affected nonimmune individuals. Secondary vaccine failures were not associated with particular MeV genotypes, and sequencing of the MeV H gene did not identify any mutation in the epitopes of hemagglutinin recognized by neutralizing antibodies that could suggest the emergence of an escape mutant.

Reduced efficacy of vaccine-induced immunity against wild type MeV strains has been hypothesized to play a role in measles reemergence [11,38]. In the case of mumps, infection in fully vaccinated individuals are not uncommon, and serum samples from vaccinated individuals have been shown to neutralize the currently circulating mumps virus genotype G less efficiently than other genotypes and the vaccine strain [39,40]. Our study demonstrated that this is not the case for measles. In fact, sera from subjects who received two doses of measles-containing vaccine cross-neutralized the infectivity of wild-type MeV isolates from the recent epidemics, including genotypes B3, D4, and D8, with the same high efficiency demonstrated against the MeV vaccine strain. Accordingly, sequencing results confirmed the antigenic stability of MeV hemagglutinin protein from different genotypes, which is the target of neutralizing antibodies, thus supporting the use of the current measles-containing vaccines. The risk of emergence of MeV mutants that escape vaccine-induced antibody neutralization is negligible. In fact, neutralizing antibodies that are present in sera of vaccinees or subjects with previous wild-type virus infection recognize immunodominant epitopes located in a region of the hemagglutinin protein that is subject to strong structural constraints, since it interacts with different receptors in human cells, such as SLAM, nectin-4, and CD46 [41].

Another factor that might contribute to the reemergence of measles in vaccinated populations could be the progressive loss of vaccine protection over time [42]. To address this concern, we measured MeV IgG and neutralizing antibody titers in subjects who received two doses of measles-containing vaccine and demonstrated the persistence of protective antibody titers up to 30 years following vaccination in over 90% of subjects, in agreement with previous studies [30]. However, vaccine-induced immunity might wane with time in the absence of boosting by exposure to wild-type virus circulation. Therefore, population immunity to measles should be monitored, especially in adult age groups, to assess potential declines of protection.

Finally, vaccine failure could be due to inborn defects in innate antiviral immunity and response to vaccination. In this regard, single-nucleotide polymorphisms of cytokine and cytokine receptor genes and genetic variants of genes involved in MeV infection, such as CD46, have been associated with measles vaccine-induced neutralizing antibodies and T cell response [43,44]. In addition, inactivating mutations in the type I interferon receptor IFNAR1 [45], the high-affinity interferon α/β receptor IFNAR2 [46], and the transcription factors signal transducer and activator of transcription (STAT) 1 [47] and STAT2 [48,49], which are involved in the control of cell responses to interferons, have been identified in children who developed disseminated infection or fatal encephalitis after inoculation of the live attenuated measles, mumps, and rubella vaccine. Thus, the role of an individual’s genetic background in vaccine responsiveness and vaccine breakthrough should be investigated.

Our study has several limitations. Seroprevalence evaluation could have been biased since it was estimated from data already available in the laboratory and did not represent homogeneously the population of the regional territory. However, it was not biased by other factors, such as refusal or non-availability to participate in an organized study [50]. For the cases of secondary vaccine failure, sera collected before infection were not available to test neutralizing and cross-neutralizing antibodies as a surrogate of protection against epidemic MeV strains. Pre-illness sera from twice-vaccinated measles cases were tested in an outbreak study in the Netherlands [51]. In that study, two out of four cases had levels of neutralizing antibodies below the estimated cutoff for clinical protection [37]. Finally, virus neutralization and cross-neutralization in the laboratory setting might not correspond to protection in vivo, even though immunological correlates of protection have been defined from clinical studies [44].

## 5. Conclusions

Measles outbreaks continue to occur in regions where vaccination coverage is below the WHO target for measles elimination. The decrease of vaccination coverage and the decline of wild-type MeV circulation pose the risk of immunity gaps in specific age groups, which should be carefully monitored in surveillance and vaccination programs. Measles-containing vaccines confer robust and sustained protection against circulating wild-type MeV strains. Sequencing of MeV genome during outbreaks showed the genetic stability of the virus, with little evolution and no positive selection pressure. Measles may occur in fully vaccinated individuals. These cases are generally characterized by a benign clinical course, low viral load, and no onward transmission and do not seem to be caused by escape mutant MeV strains. Taken together, these findings support the use of the current measles-containing vaccines. Vaccination campaigns should be promoted and enhanced to pursue the goal of measles eradication.

## Figures and Tables

**Table 1:** Evolutionary analysis on whole genomes of measles virus (MeV) B3 and D8 isolates ^1^.

Gene	dN/dS	Negatively Selected Sites (Amino Acid Position)
Genotype B3 (25 samples)		
L	0.249	1695
N	0.107	220
P	0	-
Genotype D8 (5 samples)		
H	0.643	-
L	0.318	56
N	0.839	-

^1^ The selection pressure was estimated only on genes which had enough variability for testing. No positive selection was found in any site; the reported negatively selected sites were confirmed by both fixed effects likelihood (FEL) and fast unconstrained Bayesian approximation (FUBAR) methods. Among B3 isolates, 21 single-nucleotide variants (SNVs) were identified (17 in the coding sequences), resulting in 12 synonymous and 5 non-synonymous amino acid substitutions. In MeV D8 isolates, 22 SNVs were detected (20 in the coding sequences); 8 of them were synonymous, 12 were non-synonymous.

**Table 2:** Clinical and laboratory characteristics of measles in previously immunized subjects.

Measles Vaccine Doses	Age (Years)	Years since Vaccination	Days from Rash	MeV IgM EIA Finding	MeV IgG EIA (IU/L)	MeV IgG avidity (%)	MeV RNA Pharyngeal Swab (C_T_)	MeV RNA Urine (C_T_)	MeV Strain
2	26	22	4	positive	15,000	84	ND	39	MVs/Padova.ITA/14.17/9 [B3]
2	30	26	0	negative	2100	58	24	30	MVs/Venezia.ITA/16.17/1 [B3]
2	26	22	1	borderline	12,000	82	27	28	MVs/Verona.ITA/20.17/1 [D8]
2	22	18	2	borderline	5000	81	30	ND	MVs/Padova.ITA/13.18/1 [B3]
2	13	9	1	negative	1460	69	38	39	ND
unknown	36	-	1	negative	2200	74	23	30	MVs/Padova.ITA/13.17/3 [B3]
unknown	42	-	1	negative	23,000	79	27	ND	MVs/Padova.ITA/17.17/4 [D8]
unknown	39	-	1	negative	5700	68	28	28	MVs/Padova.ITA/16.17/8 [B3]
unknown	68	-	1	negative	13,000	86	ND	27	MVs/Venezia.ITA/16.17/1 [B3]
unknown	29	-	1	borderline	21,000	81	22	18	MVs/Venezia.ITA/10.17/2 [B3]
unknown	52	-	2	positive	15,000	84	29	30	MVs/Venezia.ITA/16.17/2 [B3]

C_T_: threshold cycle of real-time RT-PCR; ND: not determined.
